# The *Lactobacillus brevis* 47 f Strain Protects the Murine Intestine from Enteropathy Induced by 5-Fluorouracil

**DOI:** 10.3390/microorganisms8060876

**Published:** 2020-06-09

**Authors:** Maria Marsova, Maya Odorskaya, Maria Novichkova, Valentina Polyakova, Serikbay Abilev, Elena Kalinina, Alexander Shtil, Elena Poluektova, Valery Danilenko

**Affiliations:** 1 Vavilov Institute of General Genetics, Russian Academy of Sciences, 119991 GSP-1 Moscow, Russia; maya_epifanova@mail.ru (M.O.); abilev@vigg.ru (S.A.); epolu@vigg.ru (E.P.); valerid@vigg.ru (V.D.); 2 Department of Biochemistry, Peoples’ Friendship University of Russia (RUDN University), 117198 Moscow, Russia; samno@yandex.ru (M.N.); kalinina_ev@rudn.university (E.K.); 3 Department of Pathological Anatomy, Orenburg Medical University, 460014 Orenburg, Russia; k_patanat@orgma.ru; 4 Blokhin National Medical Research Center of Oncology, 24 Kashirskoye shosse, 115478 Moscow, Russia; shtilaa@yahoo.com; 5 Institute of Gene Biology, Russian Academy of Sciences, 119334 Moscow, Russia; 6 Department of Chemistry, Moscow State University, 1-3 Leninskie Gory, 119991 Moscow, Russia; 7 Faculty of Ecology, International Institute for Strategic Development of Sectoral Economics, Peoples’ Friendship University of Russia (RUDN University), 117198 Moscow, Russia

**Keywords:** lactobacillus, 5-fluorouracil, mucositis, oxidative stress, antioxidants

## Abstract

We report that the results of our study indicate that *Lactobacillus brevis* 47 f strain isolated from the faeces of a healthy individual prevents the manifestations of experimental mucositis induced by treatment of Balb/c mice with the anticancer drug 5-fluorouracil (5 FU; 100 mg/kg i.p. × 3 days). The presence of damage to the intestine and the colon was determined by a morphometric analysis of specimens including the height of villi, the amount of goblet cells and infiltrating mononuclear cells, and the expression of the proliferative Ki-67 antigen. Changes in the lipid peroxidation in the blood and the intestine were determined by severalfold increase of the concentration of malonic dialdehyde. Oral administration of *L. brevis* 47 f strain prior to 5 FU decreased the drug-induced morphological and biochemical changes to their respective physiological levels; the ability of intestinal epitheliocytes to express Ki-67 was partially restored. These effects of *L. brevis* 47 f strain were more pronounced or similar to those of the reference compound Rebamipid, a quinoline derivative known to protect the gut from drug-induced toxicity. Thus, the new lactobacilli strain attenuates the severity of 5 FU-induced enteropathy.

## 1. Introduction

Reactive oxygen species (ROS) represent a key mechanism of regulation of a plethora of physiological processes. This evolutionarily-conserved mechanism regulates metabolism in all organisms, from *Protozoa* to mammals [1,2]. However, ROS elevation and an unrestricted oxidation may lead to damage of subcellular structures and cell death [3]. To combat ROS bursts for therapy a variety of antioxidants of different types have been used [4,5,6,7].

Probiotic bacteria, particularly lactobacilli, have long been recognized as a natural source of potentially useful agents. These species can be advantageous over chemical compounds due to their safety and biocompatibility. Many lactobacilli display antioxidant activity that has been determined in bacterial cultures and in animal models. This activity can be found in living as well as in heat-inactivated bacteria, in cell free extracts and in culture supernatants, indicating that individual cell components and metabolites can have antioxidative properties [6,8] Furthermore, lactobacilli produce a number of biologically active compounds such as peptides, short chain fatty acids, microRNAs, and lipoteichoic acid [9,10,11], thereby activating the antioxidant enzymes such as catalase, superoxide dismutase, and glutathione peroxidase via Nrf2-Keap1-ARE-, nuclear factor kappa B (NFκB)-, and mitogen-activated protein kinase/protein kinase C pathways [12,13]. The antioxidant properties, together with immunostimulatory and metabolic activities, and antagonism against pathogenic microorganisms, make lactobacilli attractive therapeutic tools in clinical situations.

Lactobacilli have recently emerged as a natural component of treatment in ulcerative colitis and inflammatory bowel syndrome [14,15]. In a broader sense, the link between the microbiota and cancer development and treatment is being established [9,16,17,18]. Indeed, the probiotic strains have demonstrated the ability to attenuate oxidative stress [19], thereby proving the principle of applicability for the combinations in cancer treatment regimens [20,21,22,23]. Particularly interesting is the application of probiotic strains in patients that undergo antitumor chemotherapy. For decades, 5-fluorouracil (5 FU) has remained an important component of chemotherapeutic regimens in gastrointestinal (GI) cancer [24,25,26]. Unfortunately, enteropathy (aka mucositis) is an unfavorable side effect of this drug. The mucosa in the digestive tract, from the oral cavity to the colon, is especially vulnerable to 5 FU. Drug-associated enteropathy is a serious obstacle for continuous treatment with 5 FU, causing a severe dehydration, pain, extension of intervals between the courses, and even lethality [27].

Rebamipide, a derivative of 2-(1H)-quinoline, demonstrated its ability to protect the GI tract. This agent stimulates the synthesis of prostaglandins and inhibits the production of pro-inflammatory cytokines in the gut mucosa [28,29]. In rats, Rebamipid (30–100 mg/kg gavage) attenuated the intestinal injury induced by indomethacin via prevention of de novo activation of genes involved in pro-inflammatory response and oxidative stress [30]. Rebamipid demonstrated its protective effects in several experimental models including 5 FU induced mucositis by attenuating diarrhea and preventing damage of intestinal epitheliocytes. These effects were attributable to an attenuation of 5 FU induced elevation of transforming growth factor β (TGF-beta) [31,32].

Earlier we have investigated the antioxidant properties of the culture medium conditioned by lactobacilli strains (total 81 strain) in a luminescent test system based on the genetically engineered strain *Escherichia coli* K12 MG1655 [9]. In this strain the hybrid plasmids containing a luciferase operon of *Photorhabdus luminescens* are regulated by inducible promoter regions of catalase and superoxide dismutase genes. The addition of superoxide anion (paraquat) and hydrogen peroxide stimulated the promoters, therefore the luminescence increased. The addition of antioxidants reduced luminescence. Two strains, *Lactobacillus plantarum* 90 sk and *L. brevis* 47 f, demonstrated the most robust antioxidant properties. The formulation based on *L. plantarum* 90 sk showed an anti-depressant activity [33]. The cell-free supernatant of *L. brevis* 47 f reduced the oxidizing activity of paraquat and hydrogen peroxide. The antioxidant capacity (Trolox equivalent) of *L. brevis* 47 f cell-free supernatant was 1.87, a value double the respective value for glutathione. Genes involved in metabolism of the antioxidant proteins glutathione and thioredoxin have been detected in *L. brevis* 47 f genome [9]. In this study we investigated the ability of *L. brevis* 47 f to protect the murine intestine from 5 FU induced mucositis. Rebamipid was used as a reference agent.

## 2. Materials and Methods

### 2.1. Bacterial Strain, Culture Media and Growth Conditions

The *L. brevis* 47 f strain (collection of the Laboratory of Genetics of Microorganisms at Vavilov Institute of General Genetics, Russian Academy of Sciences) was isolated from the gut biotope of a healthy female resident of Central European part of the Russian Federation. Strain identification was based on 16 S ribosomal RNA gene sequencing [34] and proved on the analysis of the entire genome (GenBank WGS LBHR01). The strain has been deposited in the All-Russian Collection of Industrial Microorganisms (No. B-12237).

The strains were grown under anaerobic conditions (10% CO_2_ atmosphere, Anaerobic System Mark II, HiMedia, Mumbai, India) at 37 °C in MRS medium (HiMedia). Standard cultures were prepared by the inoculation of 4 mL MRS broth with 10 μL of a frozen stock (−80 °C), followed by incubation at 37 °C for 20 h. Strains were then subcultured in 10 mL MRS broth for 20 h at 37 °C prior to inoculation into the fermentation vessel. To obtain the product for biological testing the bacterial culture was propagated in a fermenter under anaerobic conditions for 18–20 h up to a stationary phase (10^9^ CFU/mL), washed with sterile PBS buffer then transferred to a solution containing 1% gelatin and 10% sucrose, incubated for 24 h at −20 °C and dried in a 2.5-L Labconco freeze drier (Labconco, Kansas City, MO, USA) under a pressure of 0.42 mBar and a temperature of −52 °C for 48 h. Vials were stored at +4 °C and the viability of the lyophilizates did not change over the course of one year.

### 2.2. Animals

The Balb/c mice (males and females, 20–22 g, 10–12 weeks old) were purchased from Stolbovaya Facility in the Moscow region and kept in Blokhin National Medical Research Center of Oncology. The experiments were approved by the Research Ethics Committee at Blokhin National Medical Research Center of Oncology (November 17, 2018; project registration number 126). Mice were kept at 18–22 °C, 30–70% air humidity, 12 h light cycle. Animals received water and food ad libitum. Mice were adapted to the vivarium for 2 weeks prior to the experiments. Animals without visible abnormalities of nutritional habits, hair cover and general behavior were used in the experiments. Males and females were treated as separate cohorts.

Mice were divided into 6 groups (21 males or 21 females per each cohort). Animal cohorts and treatments are presented in Table 1. Mice in group 1 (mock-treated control) received i.p. injections of 0.3 mL saline at days 22–24. The test cohorts included group 2 (5 FU 100 mg/kg in 0.3 mL saline i.p. at days 22–24), group 3: *L. brevis* 47 f at days 1, 8 and 15 (10^8^ CFU/animal in 0.5 mL of aqueous suspension as daily gavage), group 4: *L. brevis* 47 f followed by 5 FU; group 5: Rebamipid at days 1, 8 and 15 (150 mg/kg in 0.5 mL of aqueous suspension; daily gavage), group 6: Rebamipid followed by 5 FU. Rebamipid was purchased from McLeods Pharm., India.

**Figure 1:**
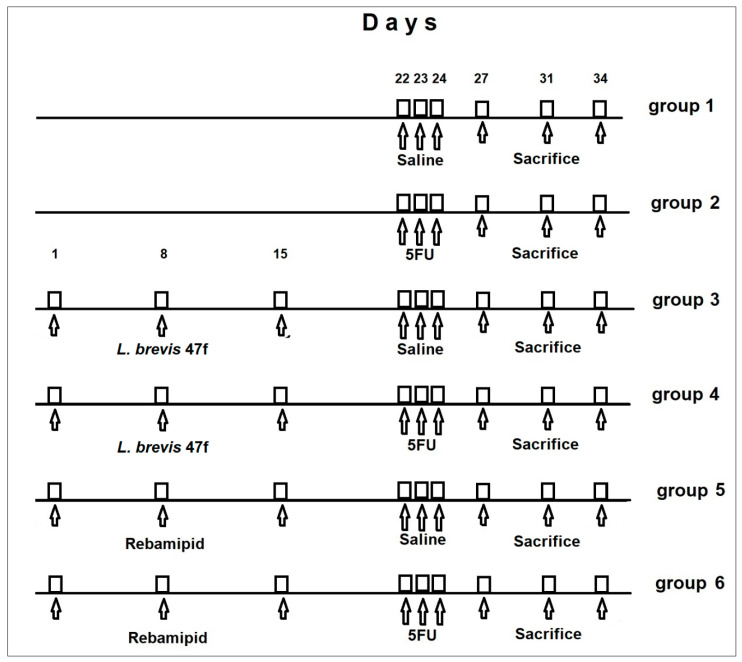
Timeline of animal treatment.

The study timeline is shown in Figure 1.

Animals were monitored for up to 10 days after the third injection of 5 FU. The total duration of the experiment was 34 days. Mice (7 animals in each group) were sacrificed at days 3, 7, and 10 after the third 5 FU injection (corresponding to days 27, 31, and 34 in Figure 1) by dislocation of cervical vertebra. The same experimental design was used for female mice. All in vivo experiments were performed in accordance with European regulation 2010/63/EU.

### 2.3. Gut Tissue Analysis by Light Microscopy

The two-to-three cm long pieces of the intestine and colon were cut, rinsed with cold saline, dried with the filter paper, weighted, then fixed in 4% buffered formaldehyde and routinely processed for hematoxylin and eosin (H&E) staining and immunohistochemistry. Morphometrical analysis included the amounts of intraepithelial lymphocytes and goblet cells per 100 epithelial cells, as well as the height of villi, calculated in ten randomly selected zones of the specimens and averaged. The Ki-67 proliferative antigen was detected with the antibody provided by Cell Signaling, (1:1000, 1 h). Secondary antibody conjugated with horseradish peroxidase was obtained from BioGenex, San Ramon, CA, USA.

### 2.4. Measurements of Malonic Dialdehyde (MDA)

Blood collected from the heart and the thoracic cavity was placed into the Eppendorf tubes containing 10% sodium citrate to prevent coagulation. The blood plasma was obtained without centrifugation and stored at 4 °C. For measurements of MDA in the tissue, one cm long fragments of the mucosa were cut, rinsed in cold saline, and homogenized in liquid nitrogen. MDA levels in the blood plasma were measured as described in [35]. Briefly, the blood sample was mixed with trichloroacetic acid (1:1 *v*/*v*), then centrifuged at 3000× *g* for 15 min, and left at 4 °C for another 15 min. The supernatant (plasma) was mixed with thiobarbituric acid (1:2 *v*/*v*), and the mixture was boiled for 15 min to develop a pink color. Samples were cooled down and centrifuged at 3000× *g* for 5 min. Optical density was measured at 535 nm in a 1 cm cuvette. The control samples containing water instead of plasma were processed exactly as above. The coefficient of molar extinction of the MDA product is ε = 1.56 × 10^5^ M^−1^ cm^−1^.

To measure MDA in the gut tissue, the homogenates were resuspended in 0.3 mL of the buffer containing 10 mM Tris base, pH 7.2 and 1 mM sodium phosphate, and left at 4 °C overnight for extraction. Then samples were centrifuged at 5000× *g* for 5 min. MDA content (µM for plasma, µmol/g of tissue) was calculated in accordance with the Lambert-Beer law, using the formula:

(1) $$C=\frac{E\times 10^{6}\times 3}{1.56\times 10^{5}},$$

where C is MDA concentration in µM; E is optical density of the sample; 10^6^ is the coefficient for calculation (µM); 3 is the dilution factor; 1.56 × 10^5^ is the coefficient of molar extinction of MDA-thiobarbituric acid complex.

### 2.5. Statistical Analysis

Comparison of animal cohorts was performed using a one-way ANOVA test with Tukey’s multiple comparisons (GraphPad Prism 8.2.0; GraphPad Software, Inc., San Diego, CA, USA). The *p* value < 0.05 between the groups was considered a statistically significant difference.

## 3. Results

### 3.1. Experimental Enteropathy Induced by 5 FU

In preliminary experiments we tested a range of 5 FU doses sufficient for the development of enteropathy. We found that 100 mg/kg i.p. caused reproducible manifestations of acute enteropathy after 3 consecutive daily injections. A reduced physical activity of animals and diarrhea were the major signs of toxicity. Hair cover was unaltered. Gross anatomical examination revealed no abnormalities in intact mice, as well as in *L. brevis* 47 f or Rebamipid cohorts. In contrast, in all 5 FU-treated animals an opaque exudate was found between thinned intestinal loops. Liquid faeces were visible in the colon’s lumen.

**Figure 2:**
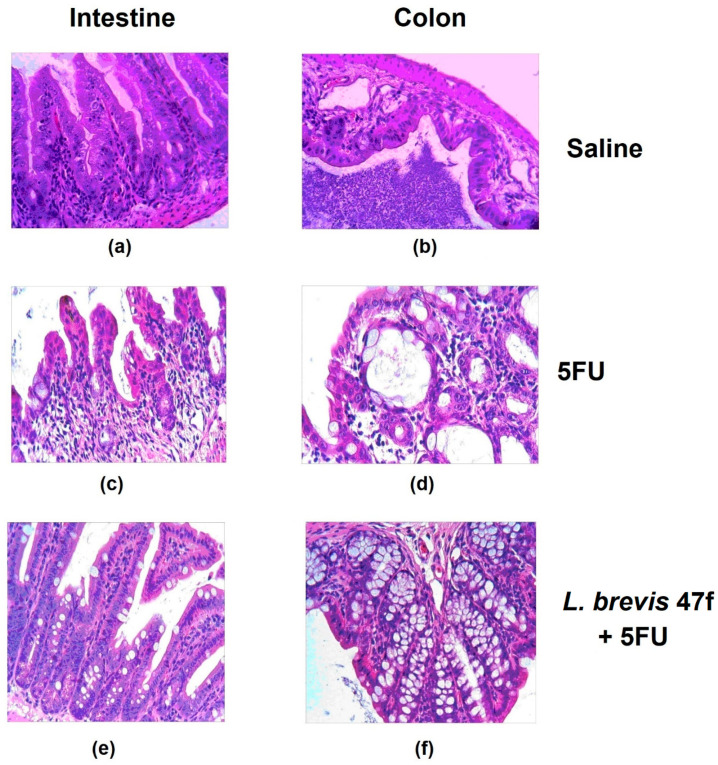
*L. brevis* 47 f prevents 5 FU-induced mucositis. (**a**,**c**,**e**): intestinal mucosa; (**b**,**d**,**f**), colon mucosa. (**a**,**b**): saline; (**c**,**d**): 5 FU 100 mg/kg daily × 3 days; (**e**,**f**): pretreatment with *L. brevis* 47 f followed by 5 FU. See *Materials and Methods* for details. Hematoxylin and eosin (H&E) staining. Initial magnification × 400.

The microscopic examination showed a dramatic damage to the intestine and the colon. The mucositis was determined by thinned epithelial layers and damaged villi (see Figure 2, comparing panels (a) and (b) with (c) and (d), respectively). *L. brevis* 47 f or Rebamipid alone evoked no effects (not shown). Importantly, *L. brevis* 47 f rescued the organ from 5 FU induced mucositis: the epithelial architectonics was largely unaltered (Figure 2, panels (e) and (f)). Rebamipid attenuated the mucositis to a similar extent as *L. brevis* 47 f (not shown).

Table 2 presents the morphometric analysis of the intestinal epithelium in mice treated with 5 FU alone or 5 FU in combination with *L. brevis* 47 f or Rebamipid. Average height of villi and the number of goblet cells were lower compared to the saline group. *L. brevis* 47 f and Rebamipid (each product alone) caused an insignificant decrease of the amount of goblet cells; a tendency to an elevated number of intraepithelial lymphocytes was observed. The height of villi remained unchanged. In the *L. brevis* 47 f + 5 FU and Rebamipid + 5 FU cohorts these parameters were close to the saline group. The number of intraepithelial lymphocytes was insignificantly smaller than in the ‘5 FU alone’ group.

**Figure 3:**
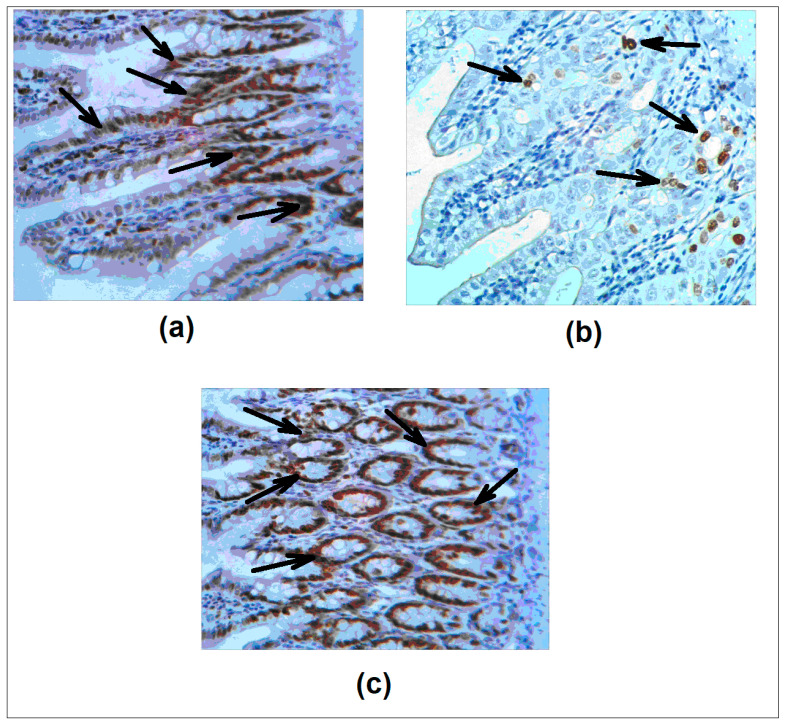
*L. brevis* 47 f restores 5 FU induced decrease of Ki-67 expression in the gut. (**a**), saline; (**b**), 5 FU; (**c**), *L. brevis* 47 f + 5 FU. Ki-67 antigen (*arrows*) was detected immunohistochemically (see *Materials and Methods*). H&E staining. Initial magnification × 400.

Next, we evaluated the Ki-67 antigen as a marker of proliferative activity of epithelial cells [36]. Figure 3a–c shows the patterns of immunohistochemical staining. Quantitative analysis revealed Ki-67 positive cells in the intestinal crypts (84.3 ± 2.3%) and in the colon (54.3 ± 3.4%). In the 5 FU cohort the percentage of proliferating cells decreased dramatically: 14.3 ± 0.4% in the intestine and 2.5 ± 0.2% in the colon. Importantly, in agreement with the net tissue protective effect of *L. brevis* 47 f, the percentages of Ki-67 positive epitheliocytes were restored: 78.4 ± 5.3% in the intestine and 50.8 ± 5.0% in the colon. Interestingly, the effect of Rebamipid was less uniform; a good level of protection was detectable in individual areas in contrast to no protection in other sites, making the statistical analysis difficult.

### 3.2. L. brevis 47 f attenuates 5 FU Induced MDA Elevation in the Intestine and the Plasma

We investigated whether the protective effect of *L. brevis* 47 f and Rebamipid on the intestine is associated with reduced peroxidation of polyunsaturated lipids, as determined by MDA levels. Table 3; Table 4 showed steady-state MDA amounts in animal cohorts in earlier (day 3) and later (days 7 and 10) time intervals after the completion of treatment. 5 FU caused a 2–2.7-fold increase of plasma MDA content compared to mock-treated mice (saline). MDA elevation by 5 FU in combination with *L. brevis* 47 f or Rebamipid was less pronounced, whereas each of these products alone did not change MDA levels.

The effects of 5 FU on MDA levels in the intestine were bigger than in the plasma. As shown in Table 5 and Table 6, 5 FU alone caused plasma MDA increase up to 4.5–13-fold, with a more robust effect in females. *L. brevis* 47 f and Rebamipid alone were inert, whereas each of these products attenuated 5 FU-induced MDA elevation. MDA levels in the intestine and plasma by day 10 after 5 FU treatment are given in Table 7.

## 4. Discussion

Normal gut microorganisms have emerged as a natural source of efficient compounds for treatment of gut inflammatory diseases, including irritable bowel syndrome, necroticizing colitis, and diverticulitis [37,38,39,40]. Lactobacilli are classical representatives of probiotic bacteria widely used in food industry [41]. Immunomodulation [42], as well as antioxidant properties [8,10,43], make lactobacilli especially attractive as pharmaceuticals.

Oral and gut mucositis remains a severe complication of otherwise efficient drug 5 FU. As proposed by van Vliet et al. [44], oxidative stress plays a pivotal role in the pathogenesis of 5 FU induced gut mucositis. Local inflammation induced by the drug stimulates intestinal permeability and alters the composition of the mucus layer, thereby allowing commensal intestinal microorganisms to overcome the tissue barrier [44]. Therefore, strategies aimed at the attenuation of 5 FU-induced ROS burst in the gut mucosa are expected to emerge as a necessary part of supportive care [45]. Different approaches have been applied to prevent drug-induced oxidative stress in the gut mucosa. For instance, regulation of Toll receptor 2 function by the agonist ligand maintains the multidrug transporter ABCB1/*MDR*1/P-glycoprotein mediated efflux and mucosal integrity [46]. In this regard, the involvement of Toll receptor/NFκB pathway emerges as an attractive therapeutic target druggable by natural compounds [47]. Indeed, attenuation of NFκB activation by the exogenous omega-3 polyunsaturated fatty acids reversed the drug-induced dysbiosis [48]. Administration of butyrate efficiently reduced 5 FU induced mucositis and intestinal damage [49]. Given that the lactobacilli strains may possess antioxidant properties, these bacteria can be a natural reservoir of products beneficial for protection from drug-induced enteropathy [50].

The antioxidant properties of *L. brevis* 47 f strain isolated from the healthy individual has been characterized in cell-free culture supernatants; also, the antioxidant genes have been identified in its genome [9]. In this study we analyzed in vivo the anti-mucositis efficacy of *L. brevis* 47 f strain, in the murine model of acute mucositis evoked by i.p. injections of 5 FU. Importantly, *L. brevis* 47 f administered as a gavage effectively protected the intestine and the colon from damage by 5 FU, as determined by gross anatomy examination and microscopic analyses. The local protective effect was concomitant with prevention of the accumulation of lipid peroxides, a marker of oxidative stress, in the intestine and the blood plasma. *L. brevis* 47 f was similarly efficacious in male and female mice. No adverse effects of the product were detectable within the time frame of experiments. Therefore the protective effect of lactobacilli was associated with attenuation of severity of symptoms of 5 FU-induced toxicity (including local mucositis and blood plasma changes). The mucosal protection by *L. brevis* 47 f was more pronounced, or similar to that of Rebamipid, a reference quinoline derivative known to protect the GI tract from pharmacologically induced oxidative stress [30,31,32].

The antioxidant properties of *L. brevis* 47 f [9,33] strain may not be the only prerequisite for the protective effect observed in the present work. Other mechanisms such as the host immune response or secretion of anti-inflammatory agents should be addressed. Furthermore, we herein showed the efficacy of *L. brevis 47 f* administered prior to 5 FU; it remains to be investigated whether the strain can be useful if the manifestations of mucositis are established. Nevertheless, the antioxidant properties, alone or in concert with other mechanisms, are important for combating rapidly developing pathological processes such as drug-induced enteropathy.

This study suggests that *L. brevis 47 f* can be applied in the functional nutrition and pharmaceutical industries. To translate our preclinical data to patient practice, the following questions remain to be resolved. A comprehensive analysis of the literature revealed that, among a variety of treatments, including sodium butyrate and dietary interventions, lactobacilli remain a major instrument for prevention of chemotherapy-induced diarrhea [22,51]. However, it is difficult to establish strict clinical guidelines due to inadequate and/or conflicting evidence. This drawback is related to variations of strains and the regimens of application, therefore efforts should be made to unify protocols. Next, the molecular mechanisms of mucosal protection should be investigated in more detail. Initial information regarding individual mechanisms [12,13,22] needs to be updated using a systematic analysis of gene expression profiles. A seminal example of such a strategy has been presented by Zhao et al. [52]. These authors employed single cell RNA sequencing to characterize the protective mechanism of alginate oligosaccharides against busulfane-induced gut mucositis in leukemia patients. Analysis of tissue samples for RNA profiling may provide valuable information about the specific patterns of mucositis which, in turn, will allow selection of the probiotic strain for personalized treatment. This strategy is expected to rationalize the use of functional nutrition in patients [53,54], as well as in healthy individuals.

Finally, our findings can be extended beyond the model analyzed in this study. Anorexia, a major symptom of chemotherapy-induced toxicity, has been attributed to a systemic dehydration due to mucositis and diarrhea in rats treated with methotrexate [55]. Interestingly, the authors demonstrated that dehydration was associated with brain perception as determined by activation of neurosecretory neurons in the hypothalamus and circumventricular areas. Thus, dehydration should be regarded as a general mechanism of chemotherapy-induced toxicity, and prevention of dehydration with properly designed patient nutrition is important for supportive care. Furthermore, the gut microbiota-brain axis has been implicated in autism spectrum disorders [56,57,58,59,60]. These data provide evidence in favor of a cooperative regulation of feeding and social behavior, therefore broadening the therapeutic potential of probiotic bacteria.

## 5. Conclusions

We demonstrated the efficacy of *L. brevis* 47 f strain in the murine model of acute mucositis evoked by i.p. injections of the anticancer drug 5 FU. Oral administration of *L. brevis* 47 f significantly attenuated the manifestations of gut mucositis induced by 5 FU in mice. The protective effects of *L. brevis* 47 f were detectable morphologically in the intestine and the colon concomitantly with prevention of lipid peroxidation in the gut tissues and in the blood plasma. The efficacy of protection by *L. brevis* 47 f was preferential over the reference compound Rebamipid. These results provide evidence to support considering *L. brevis* 47 f as a product that can be useful in supportive care for patients who undergo antitumor chemotherapy.

## Figures and Tables

**Table 1:** Animal groups and treatments. 5 FU; 5-fluorouracil.

Group	Treatment
Control (#1)	Saline × 3 days
Test (#2)	5 FU 100 mg/kg in saline × 3 days
Test (#3)	*L. brevis* 47 f 10^8^ CFU × 3 times gavage
Test (#4)	*L. brevis* 47 f 10^8^ CFU × 3 times + 5 FU 100 mg/kg × 3 days
Test (#5)	Rebamipid 150 mg/kg × 3 times
Test (#6)	Rebamipid 150 mg/kg × 3 times + 5 FU 100 mg/kg × 3 days

**Table 2:** Morphological parameters of intestinal mucosa in mice exposed to 5 FU with or without *L. brevis* 47 f or Rebamipid.

Parameter	Saline	5 FU	*L. brevis* 47 f	Rebamipid	*L. brevis* 47 f + 5 FU	Rebamipid + 5 FU
Height of villi, µm	175 ± 14	102 ± 12 *	166 ± 16	176 ± 14	149 ± 11	130 ± 16
Goblet cells/100 epitheliocytes	29 ± 8	11 ± 6 *	21 ± 6	22 ± 4	21 ± 6	20 ± 5
Intraepithelial lymphocytes/ 100 epitheliocytes	20 ± 4	27 ± 7	25 ± 3	26 ± 3	24 ± 4	23 ± 2

Mice were sacrificed at day 27 (Figure 1). * *p* < 0.05 compared to other cohorts.

**Table 3:** Time course of malonic dialdehyde (MDA) levels in the male intestine.

Group	Treatment	MDA		
		Day 3/27 *	Day 7/31	Day 10/34
1	Saline	0.7 ± 0.4	0.9 ± 0.6	0.6 ± 0.4
2	5 FU	2.4 ± 0.4	4.2 ± 0.4	4.9 ± 0.6
3	*L. brevis* 47 f t	0.7 ± 0.3	1.1 ± 0.3	1.3 ± 0.4
4	*L. brevis* 47 f + 5 FU	0.9 ± 0.3 *	1.9 ± 0.6 *	2.6 ± 0.6 *
5	Rebamipid	0.5 ± 0.2	1.3 ± 0.3	1.0 ± 0.2
6	Rebamipid + 5 FU	1.4 ± 0.3	3.3 ± 0.4	5.0 ± 0.5

Mice were sacrificed at days 3, 7 or 10 after the 3 d injection of 5 FU (days 27, 31 or 34, respectively; Figure 1). Values are mean ± SD (*n* = 7). * *p* < 0.05 compared to group 2.

**Table 4:** Time course of MDA levels in the female intestine.

Group	Treatment	MDA		
		Day 3	Day 7	Day 10
1	Saline	0.3 ± 0.1	0.320 ± 0.123	0.334 ± 0.066
2	5 FU	3.1 ± 0.4	3.3 ± 0.6	3.8 ± 0.5
3	*L. brevis* 47 f	0.4 ± 0.1	0.3 ± 0.1	0.5 ± 0.1
4	*L. brevis* 47 f + 5 FU	1.5 ± 0.3 *	1.6 ± 0.2 *	1.4 ± 0.3 *
5	Rebamipid	0.3 ± 0.1	0.4 ± 0.1	0.3 ± 0.1
6	Rebamipid + 5 FU	1.4 ± 0.3	2.4 ± 0.5	3.9 ± 0.4

Mice were sacrificed at days 3, 7 or 10 after the 3 d injection of 5 FU (days 27, 31 or 34, respectively; Figure 1). Values are mean ± SD (*n* = 7). * *p* < 0.05 compared to group 2.

**Table 5:** Time course of MDA levels in the male plasma.

Group	Treatment	Plasma MDA		
		Day 3	Day 7	Day 10
1	Saline	3.4 ± 0.5	3.2 ± 0.5	3.4 ± 0.4
2	5 FU	6.4 ± 0.8	6.2 ± 0.8	6.4 ± 0.9
3	*L. brevis* 47 f	2.6 ± 0.3	2.6 ± 0.4	3.2 ± 0.6
4	*L. brevis* 47 f + 5 FU	3.8 ± 0.7 *	3.3 ± 0.6 *	3.5 ± 0.4 *
5	Rebamipid	3.2 ± 0.3	3.1 ± 0.6	3.6 ± 0.6
6	Rebamipid + 5 FU	5.5 ± 0.5	5.7 ± 0.4	5.3 ± 0.5

Male mice were sacrificed at days 3, 7 or 10 after the 3 d injection of 5 FU (days 27, 31 or 34, respectively; Figure 1). Values are mean + SD (*n* = 7). * *p* < 0.05 compared to group 2.

**Table 6:** Time course of MDA levels in the female plasma.

Group	Treatment	Plasma MDA		
		Day 3	Day 7	Day 10
1	Saline	3.0 ± 0.4	3.4 ± 0.4	3.3 ± 0.5
2	5 FU	6.9 ± 0.5	7.0 ± 0.6	8.3 ± 0.4
3	*L. brevis* 47 f	3.2 ± 0.4	2.9 ± 0.4	3.4 ± 0.3
4	*L. brevis* 47 f + 5 FU	4.3 ± 0.5*	4.6 ± 0.4*	4.6 ± 0.3*
5	Rebamipid	2.9 ± 0.2	3.1 ± 0.4	3.5 ± 0.4
6	Rebamipid + 5 FU	6.1 ± 0.4	7.4 ± 0.5	6.1 ± 0.6

Female mice were sacrificed at days 3, 7, or 10 after the 3 d injection of 5 FU (days 27, 31 or 34, respectively; Figure 1). Values are mean ± SD (*n* = 7). * *p* < 0.05 compared to group 2.

**Table 7:** Fold increase of MDA levels by day 10 after 5 FU treatment.

Group	Treatment	Plasma		Intestine	
		Males	Females	Males	Females
1	Saline	1	1	1	1
2	5 FU	1.9	2.3	6.3	12
3	*L. brevis* 47 f	0.8	1	1.7	1.2
4	*L. brevis* 47 f + 5 FU	1.1	1.4	3.3	4.7
5	Rebamipid	1	1	1.2	1.1
6	Rebamipid + 5 FU	1.7	2	6.4	12
